# Obesity prevention and the role of hospital and community-based health services: a scoping review

**DOI:** 10.1186/s12913-019-4262-3

**Published:** 2019-07-05

**Authors:** Claire Pearce, Lucie Rychetnik, Sonia Wutzke, Andrew Wilson

**Affiliations:** 1 The Australian Prevention Partnership Centre, Sydney, NSW Australia; 2 Menzies Centre for Health Policy, University of Sydney, Sydney, NSW Australia; 3 Canberra Health Services, Canberra, ACT Australia; 4 School of Medicine, University of Notre Dame, Sydney, Australia

**Keywords:** Obesity, Prevention, Health services

## Abstract

**Background:**

Control of obesity is an important priority to reduce the burden of chronic disease. Clinical guidelines focus on the role of primary healthcare in obesity prevention. The purpose of this scoping review is to examine what the published literature indicates about the role of hospital and community based health services in adult obesity prevention in order to map the evidence and identify gaps in existing research.

**Methods:**

Databases were searched for articles published in English between 2006 and 2016 and screened against inclusion and exclusion criteria. Further papers were highlighted through a manual search of the reference lists. Included papers evaluated interventions aimed at preventing overweight and obesity in adults that were implemented within and/or by hospital and community health services; were an empirical description of obesity prevention within a health setting or reported health staff perceptions of obesity and obesity prevention.

**Results:**

The evidence supports screening for obesity of all healthcare patients, combined with referral to appropriate intervention services but indicates that health professionals do not typically adopt this practice. As well as practical issues such as time and resourcing, implementation is impacted by health professionals’ views about the causes of obesity and doubts about the benefits of the health sector intervening once someone is already obese. As well as lacking confidence or knowledge about how to integrate prevention into clinical care, health professional judgements about who might benefit from prevention and negative views about effectiveness of prevention hinder the implementation of practice guidelines. This is compounded by an often prevailing view that preventing obesity is a matter of personal responsibility and choice.

**Conclusions:**

This review highlights that whilst a population health approach is important to address the complexity of obesity, it is important that the remit of health services is extended beyond medical treatment to incorporate obesity prevention through screening and referral. Further research into the role of health services in obesity prevention should take a systems approach to examine how health service structures, policy and practice interrelationships, and service delivery boundaries, processes and perspectives impact on changing models of care.

## Background

Chronic diseases place a significant burden on the Australian healthcare system. They account for 90% of all deaths [1] and significantly reduce quality of life [2]. Being obese is a major risk factor for many chronic diseases including heart disease, cancer, kidney failure, pulmonary disease and diabetes [3, 4]. Being overweight can impede the management of chronic conditions and is the second highest contributor to burden of disease. Obesity has been shown to reduce quality-adjusted life expectancy [5].

The World Health Organisation (WHO) highlights prevention of obesity as an important priority to reduce the impact of non-communicable disease. Both supporting people who are currently overweight to attain modest weight loss as well as preventing further increases in weight may eventually see a decrease in overall rates of obesity and a reduction in the rates of chronic diseases [6] and therefore a decrease in associated costs [7].

International guidelines recommend that preventive care be provided across the whole health system, integrated into ‘curative’ or disease management focused consultations, regardless of age or health status [8]. For obesity prevention, there are specific guidelines for the role of the general practitioner, for example the Royal Australian College of General Practitioners ‘Guidelines for preventive activities in general practice’ [9]. However, the prevention role of hospital and community health services is not as clearly articulated, particularly in relation to an adult population.

In this research we present a review of published literature investigating the role of hospital and community based health services in adult obesity prevention. The aim is to improve understanding of the role for hospital and community based health services in prevention as well as the potential enablers and barriers to the delivery of preventive health services in order to inform future research to support the development of obesity prevention guidelines applicable to a range of health service settings.

## Methods

A scoping review [10] was conducted to map evidence and identify gaps in the extent, range, and nature of research undertaken in relation to the role of health services in obesity prevention. The focus of the review was on hospital and community based health services as unlike primary care, the roles of these services in obesity prevention are not clearly outlined in clinical guidelines.

### Research question

The overarching question for this scoping study was: What does the peer reviewed literature reveal about the role of adult health services (excluding general practice) in the provision of obesity prevention and what are the key elements of implementation?

### Data sources and search

Three databases (CINAHL and Medline concurrently and PubMed) were searched for references containing the words “obese” AND “prevent*” AND “healthcare/ health services” AND “adult”. Medline and CINAHL were searched concurrently to cover medical, nursing and allied health research. PubMed was searched to pick up those articles not yet assigned MESH headings. For practical reasons, the scope was limited to articles published in English between 2006 and 2016 (November). The Cochrane database was searched using the phrase “Prevention of overweight and obesity” to include systematic reviews conducted in the last 10 years.

### Inclusion and exclusion criteria

As the aim of the review was to highlight clinical interventions as well as issues relating to implementation, papers were included if they fell into any of the following categories: (1) Evaluation of a specific hospital or community health based obesity prevention intervention; (2) Clinical guidelines featuring obesity prevention; (3) Systematic or scoping reviews of health service based obesity prevention or (4) Empirical description of obesity prevention within a health setting. A fifth category was identified in the process of undertaking the review: (5) Health staff or health service consumer perceptions of and beliefs about obesity and obesity prevention. For each of these categories, the focus of the intervention was on services for adults. We included primary studies as well as literature reviews.

Articles that were excluded were those that:

- focused on prevention of childhood obesity;
- were medical treatments aimed solely at weight loss, such as surgical or pharmaceutical interventions;
- described an intervention that did not take place in a health setting or if that setting was focused solely on the role of general practitioners.

Papers were also excluded if they described obesity or associated disease but did not focus on interventions with a goal of prevention or if the focus was on population health initiatives that were not within the remit of health services, such as introducing food taxes. Opinion pieces and editorials were not included.

### Data extraction

All articles were reviewed and divided into the categories described above. Information was summarised using a standardised extraction form developed for the review (see Tables 1, 2, 3, 4, 5) to identify the clinical areas where prevention is effective and the fundamental elements of implementation.

**Table 1:** Scope of literature by category. Category 1: Evaluation of a specific hospital or community health based obesity prevention intervention

Study (author, year, country)	Clinical focus	Intervention participants, setting duration	Main findings and limitation	5As focus (Ask, Assess, Advise/Agree, Assist, Arrange)
Jackson et al., 2007 [11] United Kingdom	Health visitor (community nurse)	Specialist health visitor intervention aimed at addressing obesity 89 people with BMI > 30 Community health 1 year	- Weight, BMI, BP decreased - Self-reported diet changed (less sugar products, more fruit and vegetable) - Positive feedback from participants - Small numbers and short term follow-up	- Assess - Advise - Assist
Davis et al. 2008 [12] USA	Medical specialists (nephrology)	Education of doctors on behaviour modification, patient education, health literacy and communication 64 patient interactions observed pre and post education of doctors working in hospital based nephrology clinic Pre and post intervention evaluation	- Doctors communication improved post intervention - Patients increased recall of weight based advice - No assessment if intervention lead to patients making changes recommended. - Small numbers, all in one clinic	- Ask - Assess - Advise
Mustila et al. 2013 [13] Finland	Maternity- Prenatal care	Non randomised, individual and group counselling for women at risk of gestational diabetes Measures: development of gestational diabetes, gestational weight gain, newborn anthropometry, infant weight gain Interventions commenced at 1—17 weeks gestational weeks, follow up to infant 12 months	- Reduced gestational glucose intolerance, no changes to gestational weight gain, newborn anthropometry or infant weight gain - No long term follow-up to establish impact on childhood obesity or mother’s long-term weight	- Ask - Assess - Advise
Claesson et al. 2014 [14] Sweden	Maternity- Physical activity benefits during pregnancy	Obese women kept physical activity diaries during pregnancy and answered questionnaires looking at mental health, QoL at weeks 15 and 35 plus 11 wks post 74 physical active, 79 physically inactive	- Physical activity among obese pregnant women provides better psychological well-being and improved quality of life, but does not change weight gain - Self-reported data	- Ask
McElwaine et al. 2014 [15] Australia	Primary healthcare based nurses and allied health	Practice change intervention to increase PHC nurse and AH provision of preventive care. Non randomised two groups (intervention and control)- interviews with clients to ascertain benefit	- Increase in assessment and advice relating to risk behaviours (Ahn, Smith, & Ory, 2012), but no change in referral rates for intervention or follow-up - Highlights issues with implementation in real world settings	- Ask - Advise - Arrange (refer)
Bartlem et al. 2016 [16] Australia	Mental health	Trial to get community MH workers to increase preventive care by assessing for risk factors and referring person for intervention 12 month intervention	- Increase in assessment for nutrition risk - No significant change in practice advice or referral	Modified 5As (2As and 1R) - Ask - Advise - Refer
Wiggers et al. 2017 [17] Australia	Community-based preventative care	Practice change intervention with nursing and allied health community base staff delivering adult services over 12 months, aimed at increasing assessment, brief advice and referral for risk factors Interventions include developing policy and electronic medical record based tool; clinician and manager training; audit and feedback; implementation support	- Assessment enhanced but no significant change to rates of brief advice or referral	Modified 5As (2As and 1R) - Ask - Advise - Refer

**Table 2:** Scope of literature by category. Category 2: Evaluation of a specific health based obesity prevention intervention: LITERATURE REVIEWS

Study (type, author, year)	Clinical focus	Summary of review	Main findings and limitations	5As focus (Ask, Assess, Advise/Agree, Assist, Arrange)
Review Smith et al. 2008 [18]	Adults (pregnancy)	Review of outcomes associated with maternal obesity in pregnancy 54 articles	Describes consequences of obesity in pregnancy; psychological implications (mainly descriptive); Interventions: community based (info, groups etc) inconclusive. Individualised: not significant numbers and no long term outcomes Makes recommendations re: implications for practice- quite broad	Not specifically highlighted by review
Cochrane review Flodgren et al. 2010 [19]	Health professional change	Interventions to change the behaviour of health professionals and the organisation of care to promote weight reduction in o/o adults (RCTs) 6 RCTS- 246 health professionals and 1324 o/o pts	Limited evidence on how to organise care to include prevention None of the studies evaluated strategies aimed at changing health professionals attitudes or beliefs	N/A focused on changing health professionals behaviour
Review of reviews Kremers et al. 2010 [20]	Adults	Lit review of interventions targeting prevention of overweight and obesity in adults Looked at 46 studies evaluating interventions aimed at preventing obesity. Interventions looked at setting and target group	More success amongst programmes targeting weight loss than at preventing CV disease or improving general health status	N/A- review focussed on service specifically designed for weight management, not process for people to get into programmes
Synthesis review Kirk et al. 2012 [21]	Adults	Synthesis of obesity management evidence Systematic reviews and meta-analysis	- Highlights the value of multi-component interventions that are delivered over the longer term, and reinforces the role of health care professionals. - Currently, few health professionals are advising their patients about weight management in general, even as the prevalence of obesity increases.	Focussed on interventions i.e. Assist and arrange
Review Vuori et al. 2013 [22] USA	Physical activity in health services	Literature review (2000–2013) of ‘exercise training’ counselling delivered in health services	Health benefits to physical activity but advice re: increasing is not routinely incorporated into health encounters Focuses on physical activity in isolation, not how it can link to other lifestyle changes such as diet	N/A Focussed on outcomes not process of providing advice
Systematic review Kushner and Ryan 2014 [23]	Clinical guidelines for adults	Systematic review to describe best practice for assessment and lifestyle management of obesity	Best practice for assessment lifestyle management of obesity is - Screen all adults for overweight, with full medical history - Offer weight loss via lifestyle change support for people with BMI > 30 Does not discuss issues relating to factors such as health literacy or how to support people with reduced capacity to make lifestyle changes Does not discuss any system issues with implementation	Ask Assess (not health literacy) Advise/ agree Assist Arrange
Cochrane review Mastellos 2014 [5]	Adults	Transtheoretical model stages of change Looking at Dietary and physical exercise modification in weight loss management for overweight and obese adults 3 RCT studies, 2971 participants	Inconclusive that this model leads to sustained weight loss. The model focuses on 5 stages of change. However, did show changes to behaviour such as improved diet and physical activity. Studies didn’t tend to focus on other outcomes e.g. QoL or rates of illness	N/A- looked at outcomes of specific interventions

**Table 3:** Scope of literature by category. Category 3: Clinical Guidelines

Author	Year	Country	Title	Summary
National Health and Medical Research Council [41]	2013	Australia	Clinical practice guidelines for the management of overweight and obesity in adults, adolescents and children in Australia.	- Guidelines for management of individuals who have a body mass index (BMI) greater than 25.0 kg/m^2^ and are at risk of comorbidities - Intended for use by clinicians including general practitioners, primary health care nurses, - follow the primary care ‘5As’ framework:
Royal Australian College of General Practitioners [9]	2016	Australia	Guidelines for preventive activities in general practice. 9th edition	Aim to provide a practical approach to weight management in general practice with a focus on more intensive interventions
National Institute for Health and Care Excellence [43]	2006 (updated 2015)	UK	Obesity prevention Clinical guideline [CG43]	Outlines role of health services in increasing physical activity levels and supporting improvements in diet
Moyer, V. A., 2011 [42] USA	2012 update of 2003 recommendations	USA	Screening for and Management of obesity in Adults: U.S. Preventive Services Task Force Recommendation Statement	Recommends all adults should be screened for obesity but that how this is done will be influenced by the individual patients circumstances as well as the health setting

**Table 4:** Scope of literature by category. Category 4: Empirical description of obesity prevention within a health setting

Study (author, year, country)	Clinical focus	Study type	Main findings and limitation
Lindstrom et al., 2005 [60] Finland	Obesity and Diabetes	Description of Finnish Diabetes Prevention Study, focussing on weight management	- Obesity needs to be seen as chronic condition and focus needs to be on behaviour change - Individuals require personalised, ongoing, long-term support to make and sustain lifestyle change - Screening high risk individuals in health settings and providing obesity prevention is effective in preventing Type 2 Diabetes
Lutfiyya et al. 2008 [24] USA	Medical services for adults	Analysis of 2003 Behavioural Risk factor Surveillance Survey to ascertain whether healthy weight patients receive primary obesity prevention advice	- Only a very small proportion of healthy-weight adults received primary prevention
Ma et al. 2009 [61] USA	Medical services for adults	Analysis of data from National Ambulatory Medical Care Survey- all patient visits in 2 year period Review of data for doctor visits to look at measurements for obesity plus rates of counselling	- Highlighted number of records that had data on weight and BMI missing plus low rates of intervention for people recorded as being overweight - Data based on one visit- not possible to track if individual received advice on other visits
Aronne 2009 [62] USA	Adults- assessment and treatment of obesity	Outlines assessment and treatment of obese individuals.	- Recommends long-term behavioural therapy to achieve the lasting benefits of weight loss interventions.
Kemper 2010 [25] USA	Adults CVD risk/ BMI and need for weight loss counselling	Reviewed records from nursing lead centre against NHLBI guidelines as to whether people were told to lose weight and how appropriate this advice was.	- Small numbers, but only 12% counselled to lose weight and those that did receive advice, it wasn’t within guidelines - Patients in programme self-selected so not reflective of broader society; self-reported risk factors
Phelan 2010 [26] USA	Maternity- weight gain during pregnancy	Discusses negatives of excessive weight gain in pregnancy and interventions	- Interventions quite broad, doesn’t highlight definite solutions but does give good summary of reasons to act during pregnancy
Heslehurst 2011 [27] UK	Maternity	Broad description of shortcomings of maternity guidelines and potential issues in UK	- Recommends further research into effectiveness of intervention to support women before, during and after pregnancy
Post et al 2011 [28] USA	Medical physicians or other community based health professionals	Analysis of survey (2005–08) which included record of BMI and question re: being told about weight status by GP or other health professional and questions re self-identifying as overweight and desire to lose weight	- People told they were overweight more likely to recognise they were overweight and express desire to lose weight - Half of overweight and third of obese not told overweight - Based on self-reported recall of being provided weight advice
Ahn, Smith et al. 2012 [29] USA	Older adults (≥65 years)	Telephone and postal survey evaluating if a doctor or nurse had asked or given advice about weight, healthy diet, or physical activity Study aimed to investigate the correlates of health professional–patient discussions about body weight, healthy diet, and physical activity.	- Being moderately or severely obese, more chronic conditions, and more frequent physician visits increased the likelihood of being recognized as overweight or obese and reporting lifestyle discussions. - Based on self-reported recall of being provided weight advice
Hernandez- Boussard et al. 2012 [63] USA	Community based medical practices	Analysis of data from National Ambulatory Medical Care Survey- all patient visits in 2 year period that recorded height and weight to ascertain whether obese patients receive same preventive care as non-obese	- Obese patients received significantly less preventative exams (e.g. mammogram, pap smear etc.); less tobacco and injury prevention advice and less psychological referrals but more diet, exercise and weight reduction education. - Data based on one visit- not possible to track if individual received advice on other visits
Oken et al. 2013 [30] USA	Maternity	Interviews regarding gestational weight gain and the use of electronic medical records to support clinical decision making Obstetric clinicians from one practice Duration N/A	- Advice regarding gestational weight gain variable, may be enhanced by having clinical decision supports in electronic medical records - Small number of participants, all from one practice
Miller et al. 2014 [31] Australia	Maternity	A general discussion of reasons for including weight management in pregnancy services and reasons why this is not happening	- A very general summary - references selective research. Gives a good overview of issues but not a definitive solution. Touches on social issues but not in great detail

**Table 5:** Scope of literature by category. Category 5: Health staffs or consumers perceptions of obesity and obesity prevention

Study (author, year, country)	Clinical focus	Study type	Main findings and limitation
Brown, Stride et al. 2007 [51] UK	Primary care nurses	Patterns of clinical practice, beliefs and attitudes of primary care nurses in relation to obesity management Self-completed postal questionnaire. 4 PCTs 544 staff- 398 responses (72%)	Majority of nurses agreed -Obesity causes health problems -Patients not motivated to change but not due to lack of self-control - empathy towards patients, rewarding to work with obese -saw weight management as part of role -did not find it awkward or sensitive issue to raise but patients perceived awkwardness. Nurses did not feel effective in role -nurses with higher BMI less likely to have negative view towards obesity - Obesity an issue of lifestyle choice -very few have specific training and didn’t think had organisational support
Durant et al. 2009 [32] USA	Community health	Survey to look at patient perception of health impact of weight. Analysis looked at ethnicity 1467 surveyed	- Large disparities on racial/ethnic grounds as to whether weight seen as negative for health - Those people whose health care provider had discussed weight had better understanding of health issues
Heslehurst et al. 2011 [33]	Maternity based health professionals	Qualitative interviews with staff working in maternity services on their views of maternity services role in caring for obese women	- Health and safety issues of working with obese women has improved but more needs to be done to address psychosocial issues, to provide clinical guidelines on weight management in pregnancy and population health initiatives to prevent obesity in pregnancy.
Smith et al. 2011 [34] (UK)	Maternal obesity	Semi-structured interviews and focus groups evaluating understanding of community based maternal obesity initiatives; community service providers views on maternal obesity services and their role in prevention and management of obesity	- Current public health and community service provision lacks structured maternal obesity objectives
Gunther et al. 2012 [35] United Kingdom	Doctors and nurses in primary healthcare	Barriers and enablers of managing obesity in GP Qualitative interviews Thematic analysis 7 GPs; 7 practice nurses; 9 O/O pts	- Barriers- stigma, cost of private services, previous patient experience, health professionals not wanting to take responsibility for obesity management; lack of consistency, lack of skills, lack of NHS services i.e. found lots of barriers - Highlighted that preventative measures that concentrate on attitudes, behaviour and short-term goals can be associated with significant health benefits.
Nahm 2012 [50] USA	Nurses	Preventive health care behaviours of USA based nurses Online study asked about diet, exercise, weight, stress and preferred preventive health status	- Nurses were aware of appropriate preventive health measures but did not translate into their own self care
Leslie et al. 2013 [36]	Maternity- Gestational weight gain	Views of socially disadvantaged, O/O newly pregnant women on GWG and resources to help with this Survey at 12 week visit	- Lack of awareness of excessive GWG
Robson et al. 2013 [37] United Kingdom	Mental health- nursing role	Postal questionnaire to 585 mental health nurses to examine attitudes to physical health care	- Mental health nurses do feel they have role in giving advice on diet and exercise but not cancer screening or smoking cessation. - More positive attitudes amongst nurses who has received physical health training post registration
Schauer et al. 2014 [38]	Primary healthcare- doctors and nurses	Semi-structured interviews with 30 doctors, doctor assistants and nurses 3	- Clinicians report addressing weight with those who have weight-related chronic conditions, are established patients, or have a change in weight since the previous visit. - Most clinicians address weight in the context of managing or preventing chronic conditions. - Many clinicians base advice on their own experiences with weight.
Tol 2014 [46] Holland	Overweight or obese adults	Readiness to change and intentions round how to make change On-line questionnaire for adults overweight or obese	- Found that adults who are medically in need of weight-related care are ready to lose weight, only a few intend to use weight related care.
Kable et Al 2015 [39] Australia	Nurses	Nurses perceptions, practices and knowledge with regard to providing healthy lifestyle advice to pts. O/O 676 surveys sent, 99 returned, 79 usable (15% response rate)	- Small numbers - 68% considered healthy lifestyle advice within scope; 28% calculated body mass, 44% mentioned physical activity solutions, 25% focused on reducing calories - Knowledge about weight management was variable
McElwaine et al. 2013 [8]	Community Health	Telephone survey of people accessing community health services regarding what preventive advice they received regarding risk factors (smoking, alcohol consumption, fruit and vegetable intake and physical activity)	- Generally preventive are not opportunistically provided. - Highest rates for smoking, lowest for fruit and vegetable consumption. - Favourable view towards receiving preventive advice
Bartlem et al. 2015 [40] Australia	Mental health	Telephone interviews with community mental health service clients re: engagement in smoking, fruit and vegetable consumption, alcohol consumption and physical activity	- High prevalence of risk behaviours plus high rate of people wanting to change behaviour

### Analysis

The primary aim of analysis was to determine the main factors in delivering adult obesity prevention within a health setting. Analysis commenced with an examination of intervention type, sample size, setting and duration. Studies were then grouped into categories that were empirically derived from the type of studies identified as summarised in Tables 1, 2, 3, 4, 5. Analysis has been framed with the 5As framework [9] which is utilised as a preventative healthcare tool to identify risk factors for chronic disease. It originated as a smoking cessation tool but has been adapted for use with obesity.

## Results

### Literature search

An initial PubMed search using the search terms “obese” AND “prevent*” AND “healthcare/ health services” AND “adult”, produced 710 articles. The first 40 of these articles were screened and found to be highly irrelevant. Subsequently, the PubMed search was changed to a title search “The Role of Health Services in the Prevention of Overweight and Obesity in Adults”. This produced 240 references, which on initial scan appeared to highlight more relevant documents. CINAHL and Medline searches using the same search terms produced 584 articles which on screening appeared to hold relevant studies. The Cochrane database search resulted in 151 references.

**Fig. 1:**
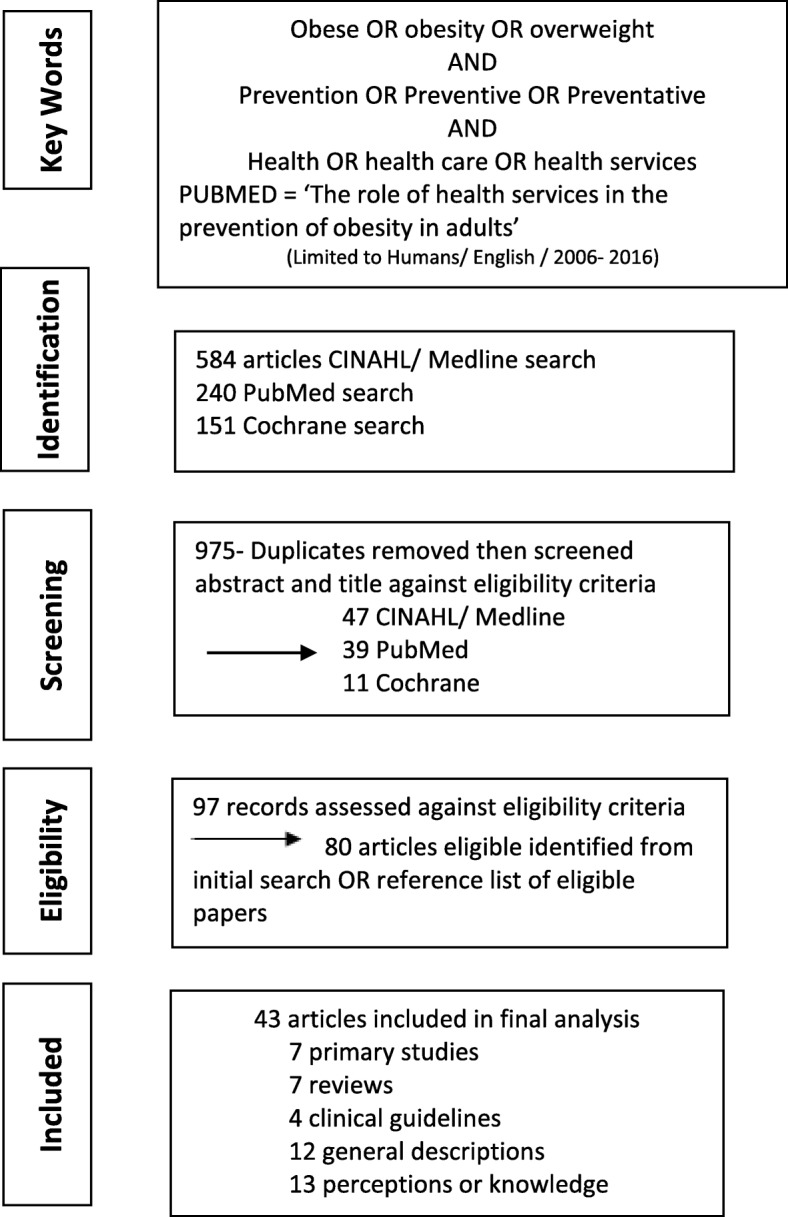
Scoping review flow chart

All references were then screened for duplicates before being assessed against the specific inclusion/ exclusion criteria. Further references were highlighted through a manual search of the reference list of those references which met the inclusion criteria. In all, 43 articles were included for review. Figure 1 presents the review flow chart.

### Scope of literature by category

Of the 43 papers included in the review, seven were primary studies of a specific health based obesity prevention intervention (Category 1) and seven were scoping or systematic reviews of specific health based obesity prevention interventions (Category 2). Four clinical guidelines were included (Category 3); two specific to the Australian context [9, 41], one from the United States [42] and one from the United Kingdom [43]. One guideline, the Royal Australian Council of General Practitioners (RACGP) Red Book [44] focussed on primary healthcare but was included as it does examine implementation of the 5As framework. This framework is frequently utilised in preventive care and though most commonly used in primary care, is one which is applicable to a range of health services. The other three focus on primary healthcare, but also consider other health services. A group of 12 papers (Category 4) provided general descriptions of obesity prevention interventions within health settings. Thirteen papers (Category 5) surveyed health professionals or consumers about their perceptions or knowledge of obesity and/or obesity prevention. A summary of the papers in each category, and the extracted data can be found in Tables 1, 2, 3, 4, 5.

### How the 5A framework informs obesity prevention

The specific health based obesity prevention interventions (Category 1 and 2), were examined using the 5As framework [44]. The 5As framework is used to identify risk factors for chronic disease, including obesity, and to plan interventions to take into account the behavioural and physiological elements to be addressed [45]. The 5As refer to Ask (about risk factors); Assess (level of risk factors, health literacy and readiness to change); Advise/ Agree (use motivational interviewing to agree goals); Assist (develop a plan to address goals) and Arrange (organise support to achieve goals and maintain change) [44].

Whilst not all the papers explicitly referred to the 5As, elements of the framework were noted in each of the seven primary studies and three of the six literature reviews concerned with health service based prevention interventions. In the section below we apply the 5A framework to consider different elements of obesity prevention and how these have been reported in the literature.

#### Ask and assess

For this review, Ask and Assess have been considered together as both focus on gathering the initial information which will determine the next step. A focus on screening is supported by evidence which shows that weighing people and discussing the risks associated with putting on excess weight has an impact on individual knowledge and readiness for change which are basic factors if obesity prevention is to be effective [36, 46]. The US Preventive Task Force and the National Heart, Lung, and Blood Institute guidelines recommend health services screen all adults for obesity [42].

Screening should include not only identifying risk factors but also ascertaining if a person is wanting to make changes to address the risk factors and their ability to do so based on factors such as health literacy, which is an individual’s ability to understand, interpret and apply information to their own health and healthcare [47]. In the included studies, there was a focus on determining risk factors but not on establishing an individual’s health literacy. The seven evaluation based papers identified a need to assess for obesity risk factors and the potential impact of these on health but only one [12] specifically concluded that there is a need to train staff in issues such as health literacy and readiness for change. This factor was missing all together from the systematic review summarising best practice in applying the framework [23].

#### Advise

All the primary study papers (Category 1) concluded that there is a role for health professionals in the provision of prevention advice and five of these seven studies discussed providing specific training to support this role [12, 13, 15–17]. However, targeted training does not automatically change practice. Two studies, one with community health staff and one with mental health clinicians, found that training changed practice in terms of assessment of risk factors but did not change practice in relation to providing advice [16, 17]. In studies which reported that clinicians did provide advice, in most cases patients could recall that advice but these papers did not report on whether the people receiving the advice changed their behaviour or on the long term retention of that advice [11–13, 15]. One systematic review [23] framed ‘advise’ in terms of telling people they needed to lose weight and how they should do that on the basis that sustained weight loss has the most significant impact on health. It did not consider supporting people to set their own goals around their weight or risk factors. The remaining six literature reviews did not report on health professionals providing advice.

#### Assist

The next step of the 5As framework is providing intervention aimed at assisting people to set goals to self-manage lifestyle changes. The primary studies (category 1) did not address this element, instead framing the role of health services not as providing support but instead referring to other agencies to provide this support. One literature review concluded that intensive long term support was required to assist people to embed changes but did not provide specific details of what this might look like [23]. Another concluded that assisting people to set goals related to weight management achieves better outcomes than linking goals to more general improvements in health [20]. The remaining literature reviews did not address the ‘assist’ element.

#### Arrange

The final step of the 5As framework recommends providing support to help people achieve and maintain their weight goals. Three of the Category 1 health service evaluations focussed specifically on this step. All were unsuccessful in increasing health professional’s rate of referral to support services. [15–17]. For example, a recent study undertaken across several community health centres focussed on supporting community health staff to incorporate assessment, brief advice and referral in relation to addressing chronic disease risk factors, including obesity risk factors. The intervention was well supported over the 12 months of implementation by a range of initiatives including pre-intervention policy change, electronic resources and staff training. The intervention was successful in getting staff to undertake more assessments for risk factors but did not change practice in relation to brief advice or referral for intervention [17]*.* Similar results were obtained within a community mental health setting, concluding that even when clinical guidelines explicitly direct clinicians to incorporate preventive care into interactions, rates of care given around issues such as fruit and vegetable intake or physical activity remain low [16]. The study concluded that prevention may need to be delivered within a different model of care [16]. Two of the systematic reviews concluded that successful obesity prevention needs to include the provision of or referral to intensive, multicomponent behavioural interventions which aim to support weight loss and management [21, 23].

### Clinical areas in which obesity prevention may be warranted

The National Health and Medical Research Council (NHMRC) Clinical Practice Guidelines [6] identify different life stages where there is a greater risk of weight gain. The empirical studies were therefore analysed to identify the clinical areas where prevention may have the most significant impact and the specific elements key to working with these clinical groups. Fifteen of the papers included in the review focused on a particular life stage or cohort of patients. The clinical areas identified were maternity, which has received the most focus but has not been rigorously evaluated [13, 14, 26, 27, 31, 33, 34, 36, 48] and mental health [37]. Definitive evidence of how obesity prevention should be delivered in mental health services was not available.

The papers which focussed on maternity based services highlight the immediate consequences of maternal obesity including higher rates of gestational diabetes, high blood pressure and pre-eclampsia and higher risk births. Excess weight gain in pregnancy combined with not losing the weight after pregnancy are predictors of long-term maternal obesity and increases the risk of the child developing obesity whilst mothers with gestational diabetes are more likely to develop type 2 diabetes later in life [36]. Along with the individual risks to mother and child, there is an increased demand for services and a requirement for more specialised services to support woman and baby both during and after the birth [18, 26, 30, 31, 33, 34].

Only one of the papers targeting obesity prevention in maternity care settings reported on a specific intervention. This found that women at risk of gestational diabetes who receive advice in relation to limiting weight gain during pregnancy are less likely to develop diabetes despite no significant difference in weight gain compared with a control group [13]. The other maternity focussed papers were more descriptive, providing a broad overview of implementation factors including the need for a multidisciplinary approach to reinforce the benefits of diet and physical activity beyond weight management. For example, obese pregnant women who are physically active have been shown to experience less depressive symptoms and report higher quality of life to obese women who are not physically active in pregnancy [14]. Two papers stated that discussions about safe weight gain and weight management needs to be done in a way that does not stigmatise or cause feelings of shame [27, 33].

Only one paper looked at a life stage other than child bearing years, namely older adults [29]. This paper summarised the results of a large survey, focussing specifically on older persons’ perceptions of receiving weight management advice. As with similar studies looking at the adult population more generally [28], it was found that older adults were more likely to receive lifestyle advice if they were already obese or had a number of chronic conditions [29]. The disadvantage of many of the survey based studies was the reliance on self-reported weight and height.

In terms of specific clinical areas, studies have been conducted in mental health and community health services. It was reported that it is very difficult to change the practice of mental health staff to include a focus of physical health risk factors [16] with mental health clinicians not necessarily seeing this as their role [37] despite the fact that people with mental illness do want to reduce their risk factors [40]. Similarly in services delivering general community health care, despite the presence of risk factors and an openness by clients to receive preventive advice, community health staff do not deliver opportunistic prevention, particularly in relation to diet [8, 17].

### Perceptions and beliefs towards obesity prevention in health services

This review found that along with practical barriers to changing practice including a lack of time, resources or clinical guidelines [34, 38, 39, 49], a key barrier to healthcare based obesity prevention is the perceptions and beliefs of health professionals towards obesity. As well as lacking confidence or knowledge about how to integrate prevention into clinical care, health professionals may simply not see it is their role [37]. There is also an issue with judgements being made in relation to who might benefit from prevention along with a negative view of the effectiveness of prevention, compounded by a view that preventing obesity is a matter of personal responsibility and choice [25, 38].

The 13 studies which specifically looked at this issue are summarised in Category 5 of Tables 1, 2, 3, 4, 5. These papers used a range of methods to ascertain attitudes, including questionnaires or surveys [8, 32, 36, 37, 39, 40, 46, 49, 50] and semi-structured interviews or focus groups [33–35, 38] and were conducted with health professionals [33–35, 37–39, 49, 50] and consumers [8, 32, 36, 40, 46]. Due to the range of methods and small numbers of many of the studies the results are not necessarily generalisable but a recurrence of themes indicates that perceptions and beliefs should be considered when incorporating obesity prevention into health care services.

The view of health professionals, that prevention is not their role, may be reinforced by the fact that they will probably not have had specific training in assessment and advice [16]. They may make judgements on who would benefit from preventive advice and tend to only raise the issue of weight if they know the patient [38]. Whilst health professionals are aware of the health implications of excess weight there may be a perception that they cannot be effective in their role due to a lack of patient motivation to enact change [25]. Other studies have shown that patients may not be told they are overweight or have the health consequences of being overweight discussed [21, 32]. This is despite evidence to suggest that being told firstly they are overweight and secondly the health risks of excess weight can impact on an individual’s readiness to make changes to diet and levels of physical activity [28]. When discussions do occur, they are more likely to be with people who are already obese [24, 28] or who have more frequent health encounters indicating that they have more complex health problems [29]. By clinicians not discussing weight and lifestyle with people before it becomes a significant problem there is a missed opportunity to prevent illness development based on known risk factors.

The uptake of prevention may also be impacted by a view that obesity is an issue of lifestyle choice and personal responsibility and therefore not the responsibility of health services unless linked to the treatment of a specific clinical condition [35, 38]. Clinical guidelines may not be consistently followed because of a lack of knowledge of the guidelines existence or a belief that the guidelines will be ineffective due to pre-conceived ideas about the group of clients being targeted or a lack of confidence in the guidelines [19, 35]*.* Specific to maternity services, clinicians acknowledge that weight gain in pregnancy is an issue but do not perceive that their patients see it as a problem [30]. In some instances, health professionals don’t feel confident talking to their patients about excess weight [35, 38, 39, 51]. These findings occur even in areas where policy is in place directing clinicians to incorporate prevention, highlighting the need for more comprehensive, multi component change management strategies to enable health professionals to develop their practice to incorporate prevention routinely into interventions [8].

Without further training, baseline knowledge on appropriate interventions to support obesity prevention is generally poor [39] and advice may be given based on the clinicians own experience of weight management [38]. Educating staff about prevention may lead to an increase in assessment of risk but not a significant increase in brief advice or referral to other services for prevention intervention [15, 17]. Both of these later elements are key to impacting on an individual’s chronic disease risk profile [16]. Training of staff may need to extend beyond principles of prevention and also include training on communicating complex information to people with low health literacy. This should include teaching techniques to ensure health professionals clarify their patient has understood information, [12] as this is a significant element in someone being able to adopt and follow preventive care advice [45].

However, the evidence of what education strategies are most effective, particularly in relation to increasing assessment and referral across all risk factors, is limited [52]. A systematic review of interventions to change the behaviour of health professionals found just six randomised control trials and the combined results of these were ambiguous [19]. When specifically looking at factors influencing health professionals decision to provide counselling regarding physical activity, the health professionals own levels of physical activity, whether or not they have specific training, knowing the patient well and the patient having risk factors for chronic disease were all influencing factors [22].

## Discussion

This review examined the literature in order to ascertain the role of hospital and community- based health services in adult obesity prevention as well as the potential enablers and barriers to the delivery of preventive health services. Whilst it is acknowledged that the health care system alone is not the answer to reducing the population impact of obesity [53], there is evidence that health services can significantly contribute to obesity prevention commencing with screening all patients for risk factors and providing brief advice. This should be followed up with referral to a service which provides long term follow-up with a focus on lifestyle change rather than just weight loss and should include consideration of an individual’s health literacy [41–44].

However, the reviewed evidence indicates that existing clinical guidelines, including the application of the 5As framework, are not being fully implemented. Where training and resources have focussed on prevention, there is an increase in the rate of screening provided but only a limited change in the rates of brief advice or referral to an intervention service [12, 15–17]. Whilst assessment of risk factors may offer some benefits, greater change is achieved when this is followed up by advice and clear, individualised input to assist people to apply the advice to their own circumstances [54].

In taking a scoping approach to the role of health services, this review was able to draw out that a significant barrier to the implementation of prevention guidelines are the perceptions of health professionals. They may not see prevention as their role [16], make judgements about the causes of and responsibility for an individual’s weight, or make subjective decisions about who will benefit from their advice [25, 35, 38]. Health professionals may also not feel sufficiently confident to raise the issue of weight because of the social meanings attached or lack of knowledge [35, 38, 39, 51]. Our review reveals these issues are common to nursing, allied health and medical staff.

Health care is predominantly delivered within a reactive model of care which is at odds with the concept of prevention [55]. Whilst there are obesity prevention guidelines which highlight the need to apply a framework such as the 5As, this fundamentally linear tool is designed to work within a traditional health care approach which focusses on the diagnosis and treatment of acute disease. As has been shown by this review, health professionals’ willingness or ability to change practice may be influenced by a range of factors, including their personal perceptions of obesity and of the potential value of prevention. So, whilst at a macro level policy and guidelines may be in place, implementation is hindered at a meso level by the mismatch between the medical model and the multifactorial causes of obesity and at a micro level by the impact of personal beliefs on patient interaction. Each of the factors dynamically influence the others so need should not be considered in isolation [53].

Changing the health system to implement effective action for the prevention of obesity therefore calls for an examination of the issues through a systems lens rather than taking a simple problem-solution driven approach. Health services are a complex system, constituted of a range of people, processes, activities, settings and structures. The interrelationships, boundaries, processes and perspectives connect in dynamic and non-linear ways which may result in emergent self-organised behaviour [56]. Importantly it should be acknowledged that systems are often nested within other systems with their own dynamics at play. Consequently, a search for solutions means identifying multiple causes as well as multiple points for intervention and being aware of unintended consequences [2, 57]. The studies identified by this review focussed on a linear approach to implementing guidelines or examined the perspectives of just one clinical team or group within a system. There is a need for research to be undertaken which, using a systems approach, examines the opportunities and threats to prevention from the perspective of a range of players within the system and considers how these perspectives might be influenced by policy and guidelines, as well as each other. This could include looking at moving beyond traditional structural boundaries to look at alternative models of care to the medical model including the use of support roles outside of those typically considered to be health professionals, particularly in the role of ongoing support [56, 58].

## Conclusions

Obesity is often described as a ‘wicked’ problem due to the multifactorial causes requiring complex solutions. Whilst a population health approach is important to address this complexity, it is important that the remit of health services is extended beyond medical treatment to incorporate obesity prevention. [59]. Though this scoping review has demonstrated that there is evidence for incorporating obesity prevention into clinical care, research to date has taken a linear approach to the implementation of guidelines without explicitly factoring in the impact of the perceptions of clinicians and managers to the prevention role or addressing the individual responsibility discourse. Further research into the role of health services in obesity prevention should take a systems approach to examine the impacts of changing models of care whilst also taking into account the perceptions of health staff towards obesity and obesity prevention and the breadth of issues impacting on each individual’s ability to make lifestyle changes.

## Strengths and limitations of the reviews

This review contributes to an understanding of the role of health services in obesity prevention by specifically focussing on services outside of primary health. The use of a scoping review allowed for broad coverage of the literature in order that the main issues could be highlighted in order to inform health policy, clinical practice and future research. The broad aims of the review may impact on attempts to replicate the review. Limiting the review to English language references may have excluded some evidence.

## Data Availability

Not applicable

## Abbreviations

NHMRC: National Health and Medical Research Council
RACGP: Royal Australian College of General Practitioners
WHO: World Health Organisation
